# ssExpression level of GAS6-mRNA influences the prognosis of acute myeloid leukemia patients with allogeneic hematopoietic stem cell transplantation

**DOI:** 10.1042/BSR20190389

**Published:** 2019-05-21

**Authors:** Xinrui Yang, Jinlong Shi, Xinpei Zhang, Gaoqi Zhang, Jilei Zhang, Siyuan Yang, Jing Wang, Xiaoyan Ke, Lin Fu

**Affiliations:** 1 Department of Hematology and Lymphoma Research Center, Peking University, Third Hospital, Beijing 100191, China; 2 Department of Biomedical Engineering, Chinese PLA General Hospital, Beijing 100853, China

**Keywords:** AML, allo-HSCT, Growth arrest-specific 6, next generation sequencing, prognosis

## Abstract

As high expression level of growth arrest-specific 6 (GAS6) had an adverse effect on prognosis in acute myeloid leukemia (AML) patients, it is interesting to reveal the relationship between GAS6-mRNA level and the survival condition of AML patients undergoing allogeneic hematopoietic stem cell transplantation (HSCT). We screened The Cancer Genome Atlas database and found 71 AML patients with GAS6-mRNA expression and received allo-HSCT treatments. We divided them into two groups based on the median expression of GAS6-mRNA. Patients with GAS6-mRNA^high^ (*n*=36) seemed to have lower bone marrow (BM) blast (*P=*0.022), lower percentage of type M5 (*P=*0.034), lower percentage of inv(16)/CBFβ-MYH11 karyotype (*P=*0.020), and lower rate of good risk classification (*P=*0.005) than the group GAS6-mRNA^low^ (*n=* 35). Higher expression level of GAS6-mRNA also brought higher RUNX1 mutations (*P=*0.003), MLL-PTD mutations (*P=*0.042), TP53 mutations (*P=*0.042), and lower NRAS/KRAS mutations (*P=*0.042). Univariate analyses showed that GAS6-mRNA was unfavorable for overall survival (OS) (*P=*0.044), as RUNX1 and WT1 also gave negative influences. Multivariate analyses confirmed that GAS6-mRNA cut down the event-free servival (EFS) and OS of AML patients with HSCT (*P=*0.029, *P=*0.025). Our study indicated that higher expression of GAS6-mRNA related with adverse effects in AML patients with HSCT treatment.

## Introduction

Characterized by clonal expansion of stem cells or progenitor cells in blood tissues without differentiation, acute myeloid leukemia (AML) is considered to be a highly heterogeneous disease [1]. As next generation sequencing was used to discover the pathogenesis of AML at the level of genes, many biomarkers for the prognosis of AML have been found. Mutations in NPM1, IDH2, and biallelic CEBPA mutations always bring longer EFS and overall survival (OS); while FLT3-ITD positive, DNMT3A, IDH1, TET2, KRAS, KIT, TP53, PTPN11, and MLL-PTD are predictors for poor outcomes [2].

Growth arrest-specific 6 (GAS6) is a gene that encodes the GAS6 protein and plays an important role in cell proliferation, survival, and migration. Since Manfioletti et al. considered GAS6 a new member of vitamin K-dependent proteins and may be involved in cell growth regulation, many studies have been done to uncover its biological function [3]. Binding with Tyro3, Axl and Mer (TAM) receptors, GAS6 gives activation for its downstream pathways like phosphatidylinositol 3-kinase (PI3K), extracellular signal-regulated kinase, and nuclear factor κ-light-chain-enhancer of activated B cells (NF-κβ) [4]. There were quite a little studies confirmed that up-regulation of GAS6 will disturb those pathways and lead to incontrollable growth of body cell and finally lead to cancer [5–8]. The prognostic value of GAS6 has been found in breast cancer, lung cancer, and some other common tumors like glioblastoma and renal cell carcinoma [9]. GAS6 expression was detected in many cell lines of leukemia [10,11]. In recent years, Whitman et al. found that GAS6 expression also produced an adverse effect on the outcome of AML patients. Expressing of GAS6 predicted CR failure, shorter DFS, and OS in patients only received chemotherapy [12]. Allo-hematopoietic stem cell transplantation (HSCT) was served as a helpful method in the recovery of AML and overcame the harmful effect of some high risk molecular biomarkers [13]. However, the prognostic significance of GAS6 expression level in AML patients undergoing allo-HSCT was still unknown. In the present study, we compared AML patients with different levels of GAS6 expression to find out whether GAS6 a poor prognosis factor in AML patients undergoing allo-HSCT.

## Materials and methods

### Patients

We screened The Cancer Genome Atlas (https://cancergenome.nih.gov/) and 71 diagnosed AML patients were enrolled in the study. Expression levels of GAS6-mRNA and clinical and molecular information of those AML patients were downloaded. We selected patients according to two standards. First, patients who do not have information about their GAS6-mRNA levels were excluded. Second, patients who did not undergo the treatment of allo-HSCT were excluded. Finally, 71 AML patients were included in our study.

Event-free survival (EFS) and OS were considered as two endpoints. EFS is the time from the date of diagnosis to removal from the study due to the absence of complete remission, relapse, or death. OS is the time from the date of diagnosis to death due to any cause. Written informed consent was obtained from all patients, which was approved by the Human Research Ethics Committee of Washington University.

### Statistical analysis

We compared the different biological and clinical characters using descriptive statistics. The Mann–Whitney U test was applied to two group comparisons, and chi-square test was used to compare the rate between two groups. Survival analysis about EFS and OS rates were calculated using the Kaplan–Meier method and compared using the log-rank test. Cox proportional hazard model was used to assess the hazard ratios (HRs) associated with the prognosis. A two-sided *P*-value <0.05 was considered statistically significant for all statistical analyses. All statistical analyses were performed by SPSS Version 20.0 software.

## Results

### Comparison of clinical and molecular characteristics between different GAS6-mRNA expression levels

Based on the median expression level, we divided 71 AML patients into two groups (GAS6-mRNA^high^, *n=* 36; GAS-mRNA^low^, *n=* 35). Clinical and molecular characteristics of two groups are summarized in Table 1 with the results of statistic analyses. No significant differences were found in age, gender, WBC count, and peripheral blood blasts proportion between two groups, while GAS6-mRNA^low^ group seemed to have higher BM blast percentage (*P=*0.022). GAS6-mRNA^low^ group was more commonly seen in type M5 when considered FAB classification (*P=*0.034). Karyotype and risk distribution showed that patients with GAS6-mRNA^high^ always have lower proportion of inv(16)/CBFβ-MYH11 karyotype (*P=*0.020) and the rate of Good Risk classification (*P=*0.005). When comparing some of the frequent AML mutations, no significant differences were observed in FLT3-ITD, NPM1, CEBPA, DNMT3A, IDH1, IDH2, WT1, TET2, KIT, PTPN11, and PHF6 between two groups, but there were obvious distinctions between those two groups as higher level of GAS6-mRNA brought higher RUNX1 mutations (*P=*0.003), MLL-PTD mutations (*P=*0.042), TP53 mutations (*P=*0.042), and lower NRAS/KRAS mutations (*P=*0.042). Relapse rate and HSCT types distribution did not show significant differences.

**Table 1:** Clinical and molecular characteristics of GAS6-mRNA^high^ and GAS6-mRNA^low^ patients

Characteristics	GAS6-mRNA^high^ (*n=* 36)	GAS6-mRNA^low^ (*n=* 35)	*U/χ^2^*	*P*-value
Age/years, median (range)	53.5 (18–69)	48 (22–72)	542.5*	0.314
Age group/n (%)			1.610^§^	0.205
<60 years	24 (66.7)	28 (80.0)		
≥60 years	12 (33.3)	7 (20.0)		
Gender/n (%)			0.010^§^	0.919
Male	21 (58.3)	20 (57.1)		
Female	15 (41.7)	15 (42.9)		
WBC count/×10^9^/l, median (range)	23.35 (0.6–223.8)	30.9 (2.3–118.8)	544.0*	0.323
BM blasts/%, median (range)	62 (30–100)	77 (34–99)	431.0*	0.022
PB blasts/%, median (range)	60 (0–96)	45 (4–94)	554.5*	0.499
FAB subtypes/n (%)				
M0	5 (13.9)	4 (11.8)	0.070^§^	0.791
M1	13 (36.1)	10 (29.4)	0.356^§^	0.551
M2	10 (27.8)	8 (23.5)	0.165^§^	0.684
M3	0 (0.0)	1 (2.9)	1.074^§^	0.300
M4	6 (16.7)	7 (20.6)	0.178^§^	0.673
M5	0 (0.0)	4 (11.8)	4.492^§^	0.034
M6	1 (2.8)	0 (0.0)	0.958^§^	0.328
M7	1 (2.8)	0 (0.0)	0.958^§^	0.328
Karyotype/n (%)				
Normal	18 (51.4)	14 (40.0)	0.921^§^	0.337
Complex	7 (20.0)	4 (11.4)	0.971^§^	0.324
8 Trisomy	5 (14.3)	1 (2.9)	2.917^§^	0.088
inv(16)/CBFβ-MYH11	0 (0.0)	5 (14.3)	5.385^§^	0.020
11q23/MLL	1 (2.9)	2 (5.7)	0.348^§^	0.555
-7/7q-	1 (2.9)	2 (5.7)	0.348^§^	0.555
t(15;17)/PML-RARA	0 (0.0)	1 (2.9)	1.014^§^	0.314
t(9;22)/BCR-ABL1	1 (2.9)	1 (2.9)	0.000^§^	1.000
t(8;21)/RUNX1-RUNX1T1	0 (0.0)	1 (2.9)	1.014^§^	0.314
Others	2 (5.7)	4 (11.4)	0.729^§^	0.393
Risk/n (%)				
Good	0 (0.0)	7 (20.0)	7.778^§^	0.005
Intermediate	23 (65.7)	17 (48.6)	2.100^§^	0.147
Poor	12 (34.3)	11 (31.4)	0.065^§^	0.799
*FLT3-ITD*			0.045^§^	0.832
Presence	9 (25.0)	8 (22.9)		
Absence	27 (75.0)	27 (77.1)		
*NPM1*			2.911^§^	0.088
Mutation	6 (16.7)	12 (34.3)		
Wild type	30 (83.3)	23 (65.7)		
*CEBPA*				
Single mutation	2 (5.6)	3 (8.6)	0.247^§^	0.620
Double mutation	2 (5.6)	1 (2.9)	0.319^§^	0.572
Wild type	32 (88.9)	31 (88.6)	0.002^§^	0.966
*DNMT3A*			0.119^§^	0.730
Mutation	8 (22.2)	9 (25.7)		
Wild type	28 (77.8)	26 (74.3)		
*IDH1*			0.002^§^	0.962
Mutation	5 (13.9)	5 (14.3)		
Wild type	31 (86.1)	30 (85.7)		
*IDH2*			0.002^§^	0.966
Mutation	4 (11.1)	4 (11.4)		
Wild type	32 (88.9)	31 (88.6)		
*WT1*			0.002^§^	0.966
Mutation	4 (11.1)	4 (11.4)		
Wild type	32 (88.9)	31 (88.6)		
*RUNX1*			8.765^§^	0.003
Mutation	8 (22.2)	0 (0.0)		
Wild type	28 (77.8)	35 (100.0)		
*MLL-PTD*			4.121^§^	0.042
Presence	4 (11.1)	0 (0.0)		
Absence	32 (88.9)	35 (100)		
*NRAS/KRAS*			4.121^§^	0.042
Mutation	1 (2.8)	6 (17.1)		
Wild type	35 (97.2)	29 (82.9)		
*TET2*			1.120^§^	0.290
Mutation	1 (2.8)	3 (8.6)		
Wild type	35 (97.2)	32 (91.4)		
*TP53*			4.121^§^	0.042
Mutation	4 (11.1)	0 (0.0)		
Wild type	32 (88.9)	35 (100.0)		
*KIT*			3.222^§^	0.073
Mutation	0 (0.0)	3 (8.6)		
Wild type	36 (100)	32 (91.4)		
*PTPN11*			0.247^§^	0.620
Mutation	2 (5.6)	3 (8.6)		
Wild type	34 (94.4)	32 (91.4)		
*PHF6*			1.001^§^	0.317
Mutation	3 (8.3)	1 (2.9)		
Wild type	33 (91.7)	34 (97.1)		
Relapse			0.065^§^	0.799
Yes	24 (68.6)	23 (65.7)		
No	11 (31.4)	12 (34.3)		
HSCT				
Haplo	1 (2.8)	1 (2.9)	0.000^§^	0.984
Sib allo	16 (44.4)	14 (40.0)	0.144^§^	0.705
MUD	19 (52.8)	20 (57.1)	0.137^§^	0.712

Allo, allogeneic; BM, bone marrow; FAB, French American British; Haplo, haploidentical; HSCT, hematopoietic stem cell transplantation; PB, peripheral blood; MUD, matched unrelated donor; WBC, white blood cell.

*Mann–Whitney *U* test. §Chi-square test.

**Figure 1:**
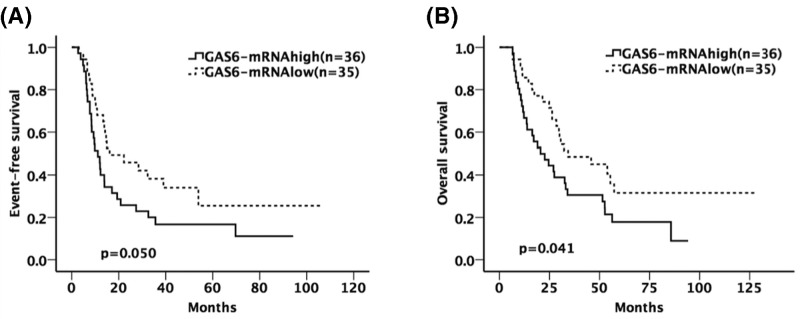
The influence of GAS6-mRNA expression on EFS and OS (**A,B**) The prognostic difference between GAS6-mRNA^high^ and GAS6-mRNA^low^ group. The group of patients with GAS6-mRNA^high^ has shorter EFS and OS than patients in the GAS6-mRNA^low^ group through underwent allo-HSCT treatment.

The group of patients with GAS6-mRNA^high^ has shorter EFS and OS than patients in the GAS6-mRNA^low^ group through underwent allo-HSCT treatment (*P=*0.050 for EFS, *P=*0.041 for OS, Figure 1A,B).

### Univatiate and multivariate analyses for prognostic factors

We assessed the prognostic factors of clinical and molecular characteristics by choosing expression level of GAS6-mRNA (high vs low), age (<60 vs ≥60 years), WBC count (<30 × 10^9^/l vs ≥30 × 10^9^/l), risk classification (poor vs non-poor), and genes with more than five mutation cases (FLT3-ITD; positive vs negative; NPM1, DNMT3A, IDH2, IDH1, RUNX1, CEBPA, WT1, PTPN11, and NRAS/KRAS; mutated vs wild) to do survival analysis. Results were shown in Table 2.

**Table 2:** Univariate analysis for EFS and OS

Variables	EFS		OS	
	OR (95% CI)	*P*-value	OR (95% CI)	*P*-value
GAS6-mRNA (high vs low)	1.727 (0.993–3.002)	0.053	1.764 (1.016–3.063)	0.044
Age (≥60 vs <60 years)	0.995 (0.545–1.816)	0.987	1.406 (0.769–2.571)	0.268
WBC (≥30 vs <30 × 10^9^/l)	1.342 (0.776–2.319)	0.293	0.986 (0.571–1.702)	0.959
Risk (poor vs non-poor)	1.081 (0.602–1.939)	0.795	1.290 (0.719–2.313)	0.393
*FLT3-ITD* (positive vs negative)	1.798 (0.951–3.398)	0.071	1.666 (0.884–3.139)	0.114
*NPM1* (mutated vs wild)	0.799 (0.419–1.523)	0.495	0.805 (0.422–1.536)	0.510
*DNMT3A* (mutated vs wild)	1.120 (0.596–2.105)	0.726	1.259 (0.668–2.374)	0.477
*IDH2* (mutated vs wild)	0.678 (0.269–1.172)	0.411	0.995 (0.392–2.525)	0.992
*IDH1* (mutated vs wild)	0.780 (0.351–1.736)	0.543	0.756 (0.340–1.678)	0.491
*RUNX1* (mutated vs wild)	1.648 (0.771–3.519)	0.197	2.437 (1.127–5.270)	0.024
*CEBPA* (mutated vs wild)	0.822 (0.326–2.075)	0.679	0.695 (0.276–1.749)	0.439
*WT1* (mutated vs wild)	2.298 (1.021–5.173)	0.045	1.587 (0.709–3.554)	0.261
*PTPN11* (mutated vs wild)	0.695 (0.275–1.756)	0.442	0.496 (0.195–1.258)	0.140
*NRAS/KRAS* (mutated vs wild)	0.878 (0.347–2.219)	0.783	1.412 (0.560–3.559)	0.465

Univariate analyses suggested that high expression of GAS6-mRNA was unfavorable for OS (*P=*0.044). Referring to common genes which always present in AML patients, RUNX1 (*P=*0.024 for OS) and WT1 (*P=*0.045 for EFS) also gave negative influences.

Then we selected above-mentioned factors that had statistical significance in univariate analyses and genes confirmed to be associated with prognosis to do the multivariate COX regression analyses (Table 3). The results indicated that high expression of GAS6-mRNA was an independent factor for poor EFS and OS (*P=*0.029, *P=*0.025) as FLT3-ITD positive (*P=*0.029, *P=*0.030).Mutations in WT1 contributed to shorter EFS (*P=*0.014), PTPN11 mutations led to shorter OS (*P=*0.007) while NPM1 mutations made longer OS (*P=*0.019). Other factors had no association with EFS and OS.

**Table 3:** Multivariate analysis for EFS and OS

Variables	EFS		OS	
	OR (95% CI)	*P*-value	OR (95% CI)	*P*-value
GAS6-mRNA (high vs low)	1.890 (1.066–3.353)	0.029	1.934 (1.086–3.441)	0.025
*FLT3-ITD* (positive vs negative)	2.382 (1.095–5.181)	0.029	2.366 (1.089–5.140)	0.030
*NPM1* (mutated vs wild)	0.479 (0.196–1.167)	0.105	0.320 (0.124–0.827)	0.019
*DNMT3A* (mutated vs wild)	1.276 (0.652–2.499)	0.476	1.379 (0.694–2.740)	0.359
*IDH2* (mutated vs wild)	0.615 (0.235–1.608)	0.321	0.996 (0.380–2.607)	0.993
*IDH1* (mutated vs wild)	1.025 (0.402–2.611)	0.959	1.170 (0.457–2.996)	0.744
*CEBPA* (mutated vs wild)	0.606 (0.224–1.636)	0.323	0.622 (0.239–1.618)	0.330
*WT1* (mutated vs wild)	3.107 (1.258–7.675)	0.014	1.959 (0.811–4.733)	0.135
*PTPN11* (mutated vs wild)	2.168 (0.695–6.764)	0.183	5.053 (1.546–16.513)	0.007
*NRAS/KRAS* (mutated vs wild)	1.311 (0.492–3.489)	0.588	0.953 (0.358–2.541)	0.924

## Discussion

Our study showed that high expression level of GAS6-mRNA has a negative effect on EFS and OS in AML patients underwent allo-HSCT treatments. Multivariate analyses also suggested that GAS6-mRNA expression level of a valuable biomarker relates to prognosis. This conclusion was in accordance with Whitman et al. whose study found that GAS6 expression caused an adverse effect on the outcome of AML patients [12]. For further thought, it indicated that allo-HSCT cannot overcome the harmful effect of GAS6-mRNA expression as well.

Mutations in NPM1 is a favorable risk factor, while FLT3-ITD positive and DNMT3A mutations are predictors for poor outcomes in AML patients [2]. In our study, univariate analyses showed that NPM1, FLT3-ITD, and DNMT3A mutations had nothing to do with EFS and OS of those patients. Multivariate analyses reached the conclusion that only the expression level of GAS6-mRNA and FLT3-ITD positive made a difference in both EFS and OS. With the ideas above, it would be reliable for us to speculate that allo-HSCT can only neutralize part of the bad effects of those traditional molecular biomarkers, but the adverse prognostic impact of GAS6-mRNA expression level still could not be reversed. Thus, the expression level of GAS6-mRNA could be a better prognostic factor for AML patients undergoing allo-HSCT compared with traditional prognostic factors.

Whitman et al. did a GAS6-associated gene expression signature and found that the overexpression of genes that relevant to cell cycle and activating of IL-8 signaling pathway were most likely to be the decisive reasons that GAS6-mRNA could have its influence on the AML patients [12]. Recent studies have found that GAS6/TAM interaction plays an important part not only in tumor cells for its biological functions, but also have a marked impact on tumor microenvironment and cancer metastasis [14]. GAS6 could promote cellular survival and down-regulate apoptotic factors [15,16], induce cell proliferation [17–19], and enhance the migration of cancer cells [20–22]. GAS6 even exerts an autocrine activity and associates with self-sustaining [23,24]. GAS6/TAM also changes the biological behavior of immune cells and vascular smooth muscle cells [25,26].

The biological role of GAS6 suggested a possibility for targetted treatments. Several studies considered that specific therapy targets for Axl R428 and non-specific therapy targets for Mer shRNA might be of use in AML patients [27,28]. These would cast new light on the treatments for AML patients with GAS6-mRNA expression.

## Conclusion

In conclusion, our study indicated that high expression of GAS6-mRNA correlates with shorter EFS and OS in AML patients with allo-HSCT treatment and it could serve as a biomarker for poor prognosis. There were several limitations in our study. The limitation of case number reduced the accuracy of our statistic process. Our study is a retrospective study, whose effectiveness is not better than a prospective study. Further studies with a larger cases number shall be done to validate our findings.

## Ethics committee approval and patient consent

Written informed consent was obtained from all patients, which was approved by the Human Research Ethics Committee of Washington University.

## Abbreviations

AML: acute myeloid leukemia
BM: bone marrow
EFS: event-free servival
GAS6: growth arrest-specific 6
HSCT: hematopoietic stem cell transplantation
OS: overall survival
